# ResFungi: A Novel Protein Database of Antifungal Drug Resistance Genes Using a Hidden Markov Model Profile

**DOI:** 10.1021/acsomega.4c02198

**Published:** 2024-07-04

**Authors:** Daniel Santana de Carvalho, Rafael Wesley Bastos, Luana Rossato, Nalu Teixeira de Aguiar Peres, Daniel Assis Santos

**Affiliations:** † Departamento de Microbiologia, Instituto de Ciências Biológicas, Universidade Federal de Minas Gerais, 31270-901 Belo Horizonte, Minas Gerais, Brazil; ‡ Bioscience Center, Federal University of Rio Grande do Norte, 59064-741 Natal, Brazil; § Faculdade de Ciências da Saúde, Universidade Federal da Grande Dourados, 79825-070 Dourados, Brazil

## Abstract

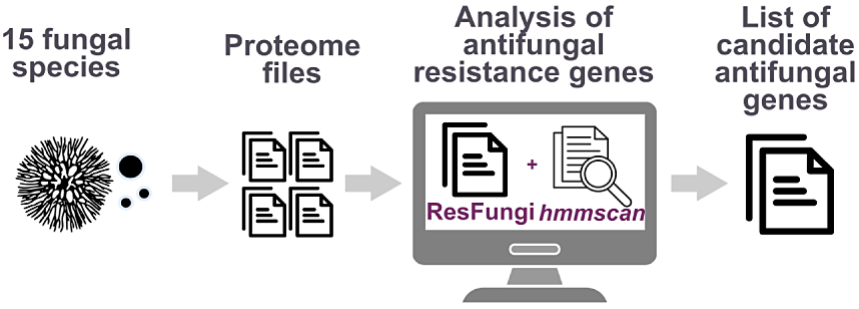

Fungal infections vary from superficial to invasive and can be life-threatening in immunocompromised and healthy individuals. Antifungal resistance is one of the main reasons for an increasing concern about fungal infections as they become more complex and harder to treat. The fungal “omics” databases help us find drug resistance genes, which is of great importance and extremely necessary. With that in mind, we built a new platform for drug resistance genes. We added seven drug classes of resistance genes to our database: azoles (without specifying which drug), fluconazole, voriconazole, itraconazole, flucytosine, micafungin, and caspofungin. Species with known resistance genes were used to validate the results from our database. This study describes a list of 261 candidate genes related to antifungal resistance, with several genes displaying transport functions involved in azole resistance. Over 65% of the candidate genes found were related to at least one type of azole. Overall, the candidate genes found have functional annotations consistent with genes or enzymes that have been linked to antifungal resistance in previous studies. Also, candidate antifungal resistance genes found exhibit functional annotations consistent with previously described resistance mechanisms. The existence of an HMM profile focusing on antifungal resistance genes allows *in silico* searches for candidate genes, helping future wet lab experiments, and hence, reducing costs when studying candidate antifungal genes without prior knowledge of the species or genes. Finally, ResFungi has proven to be a powerful tool to narrow down candidate antifungal-related genes and unravel mechanisms related to resistance to help in the design of experiments focusing on the genetic basis of antifungal resistance.

## Introduction

Drug-resistant fungal infections are an emerging threat, especially in immunocompromised patients. Also, fungal infections are difficult to diagnose and become dangerous when it is an invasive and disseminated disease.^1,2^ In recent years, the number of studies regarding drug resistance in fungi have been increasing, with the description and identification of mutations in genes that confer antifungal resistance to several drugs.^3−6^

Among the critical group in the fungal priority pathogen list published by the World Health Organization are the yeasts: *Candida albicans*, *Candida auris*, and *Cryptococcus neoformans*.^7,8^*Candida* species have been targeted as a serious pathogen due to their increasing worldwide spread and multidrug resistance. *Candida albicans* and *Candida auris* strains resistant to fluconazole have been widely isolated,^9^ and voriconazole resistance has also been described for *C. albicans*.^10,11^ Moreover, multidrug-resistant *C. auris* isolates have already been described.^12,13^*Cryptococcus neoformans* causes a serious fungal disease associated with a high mortality rate in immunocompromised individuals. *C. neoformans* exhibiting azole resistance has also been described.^14^ Overall, studies that use genomic data to predict antifungal resistance are still unavailable.

With the advances in DNA sequencing, several databases with fungal “omics” information have been developed, such as FungiDB (“omics” database),^15^ MG-RAST (metagenomics database),^16^ fungal genome initiative,^17^ and Mycology Antifungal Resistance Database (MARDy).^18^ These databases enable high-throughput genomic, transcriptomic, and proteomic analyses. Moreover, protein family databases are crucial for determining protein function in different organisms based on sequence homology, which helps to understand the mechanisms involved in drug resistance. Here, we propose a novel database that focuses on antifungal resistance genes that can be used to predict resistance genes in species without available drug resistance data. Protein databases focusing on the prediction of antifungal resistance from genomic data are not yet available, which makes the construction of such a database relevant for studies where no prior knowledge of resistance genes or mechanisms is described in the literature.

The basic local alignment search tool (BLAST) is a powerful method to search for nucleotide and protein sequence homology;^19^ however, fast-evolving sequences can present as a challenge to find homologs.^20^ Environmental conditions can lead to increased mutation rates in fungal genomes which help them to survive under stress conditions and develop de novo resistance to antifungal drugs.^21^ For this reason, BLAST searches might fail to retrieve homologous sequences that confer antifungal resistance genes and protein sequences. Therefore, it is important to use different strategies to overcome this challenge.

One way to work with protein data to search for drug resistance genes is using hidden Markov models (HMMs). The profiles of HMMs are very useful to improve multiple sequence alignments,^22^ but they can be computationally expensive and time-consuming. In 2011, the software HMMER3 was able to overcome some of the computational limitations of using HMMs to find sequence homology, making the process much faster.^23^ HMMER3 can also be used to generate profile HMMs from multiple sequence alignments as well as to search for homologous sequences. Interestingly, HMM protein profiles can be found for antibiotic resistance genes in bacteria, for instance, Resfams^24^ and AMRFinderPlus,^25^ allowing studies that describe bacterial resistomes. However, resistome predictors for fungi are not yet available.^8^

Here, we describe a novel HMM profile database with previously validated antifungal resistance genes obtained from previously published studies and from MARDy.^18^ To validate the ResFungi database, we performed a sequence homology search using HMMER3^23^ for analyzing species with known resistance genes to the drugs included in the database. Here, we were able to retrieve 261 candidate genes among 15 fungal species from different groups. The results show that ResFungi retrieved known antifungal resistance genes as well as predicted other genes that might confer antifungal resistance.

## Material and Methods

### Obtaining Known Antifungal Resistance Genes

The antifungal resistance genes were obtained from the Mycology Antifungal Resistance Database (MARDy) website (http://mardy.dide.ic.ac.uk/).^18^ The database was downloaded sorting results by drugs; that is, genes from different species were grouped by mutations that confer resistance to a certain drug. The gene IDs from the database were used to retrieve the protein sequences of each gene in different species. Only drugs that had at least three sequences documented were selected to build the protein family HMM. The choice to generate HMM profiles only for drugs with at least three sequences was to minimize weak analyses of conserved domains and to be able to generate profiles despite the limited number of antifungal resistance genes described in the literature. To increase the gene list obtained from MARDy, resistance genes recently published for *Cryptococcus* species^26−28^ and *A. fumigatus* Af293^29^ were also included. After the list of genes that conferred resistance to different drugs was obtained, gene sequences were downloaded manually from he NCBI and saved to different fasta files based on the drugs they conferred resistance to.

### Generating the Hidden Markov Model (HMM) profile

Each fasta file with the sequences, created for each drug, was aligned using Muscle version 5.1^30^ with default parameters. The aligned fasta files were used to generate the HMM profiles using the HMMER3^31^*hmmbuild* function with default parameters. Once the protein family HMMs were generated, tests were performed with fungal species known to exhibit drug-resistant mutations (*C. albicans*, *C. auris*, and *C. neoformans*). The protein sequences in the ResFungi database are related to resistance to azoles, caspofungin, fluconazole, itraconazole, flucytosine, micafungin, and voriconazole.

### Obtaining Proteomes for Case Studies

A list of 15 species representing fungi with medical, animal, or environmental importance was used as a case study to assess ResFungi’s ability to find candidate antifungal resistance genes. The following species had their genome and proteome data obtained from public databases: *Candida albicans* SC5314 (https://www.ncbi.nlm.nih.gov/datasets/genome/GCF_000182965.3/), *Candida auris* B8441 (http://www.candidagenome.org/download/sequence/C_auris_B8441/current/), *Cryptococcus neoformans* var. *grubii* H99 (https://fungi.ensembl.org/Cryptococcus_neoformans_var_grubii_h99_gca_000149245/Info/Index), *Aspergillus fumigatus* Af293 (https://www.ncbi.nlm.nih.gov/datasets/taxonomy/746128/), *Aspergillus terreus* NIH2624 (https://www.ncbi.nlm.nih.gov/datasets/genome/GCF_000149615.1/), *Sporothrix brasiliensis* 5110 (https://www.ncbi.nlm.nih.gov/datasets/genome/GCF_000820605.1/), *Sporothrix schenckii* ATCC 58251 (https://www.ncbi.nlm.nih.gov/datasets/genome/GCA_000474925.1/), *Histoplasma capsulatum* G186AR (https://www.ncbi.nlm.nih.gov/datasets/genome/GCF_000150115.1/), *Coccidioides immitis* H538.4 (https://www.ncbi.nlm.nih.gov/datasets/genome/GCA_000149815.1/), *Paracoccidioides brasiliensis* Pb03 (https://www.ncbi.nlm.nih.gov/datasets/genome/GCA_000150475.2/), *Trichophyton rubrum* CBS 118892 (https://www.ncbi.nlm.nih.gov/datasets/genome/GCF_000151425.1/), *Microsporum canis* CBS 113480 (https://www.ncbi.nlm.nih.gov/datasets/genome/GCF_000151145.1/), *Fusarium graminearum* PH-1 (https://www.ncbi.nlm.nih.gov/datasets/genome/GCF_000240135.3/), *Exophiala mesophila* CBS 40295 (https://www.ncbi.nlm.nih.gov/datasets/genome/GCF_000836275.1/), and *Fonsecaea multimorphosa* CBS 102226 (https://www.ncbi.nlm.nih.gov/datasets/genome/GCF_000836435.1/). The species analyzed represent seven distinct fungal orders.

**Figure 1:**
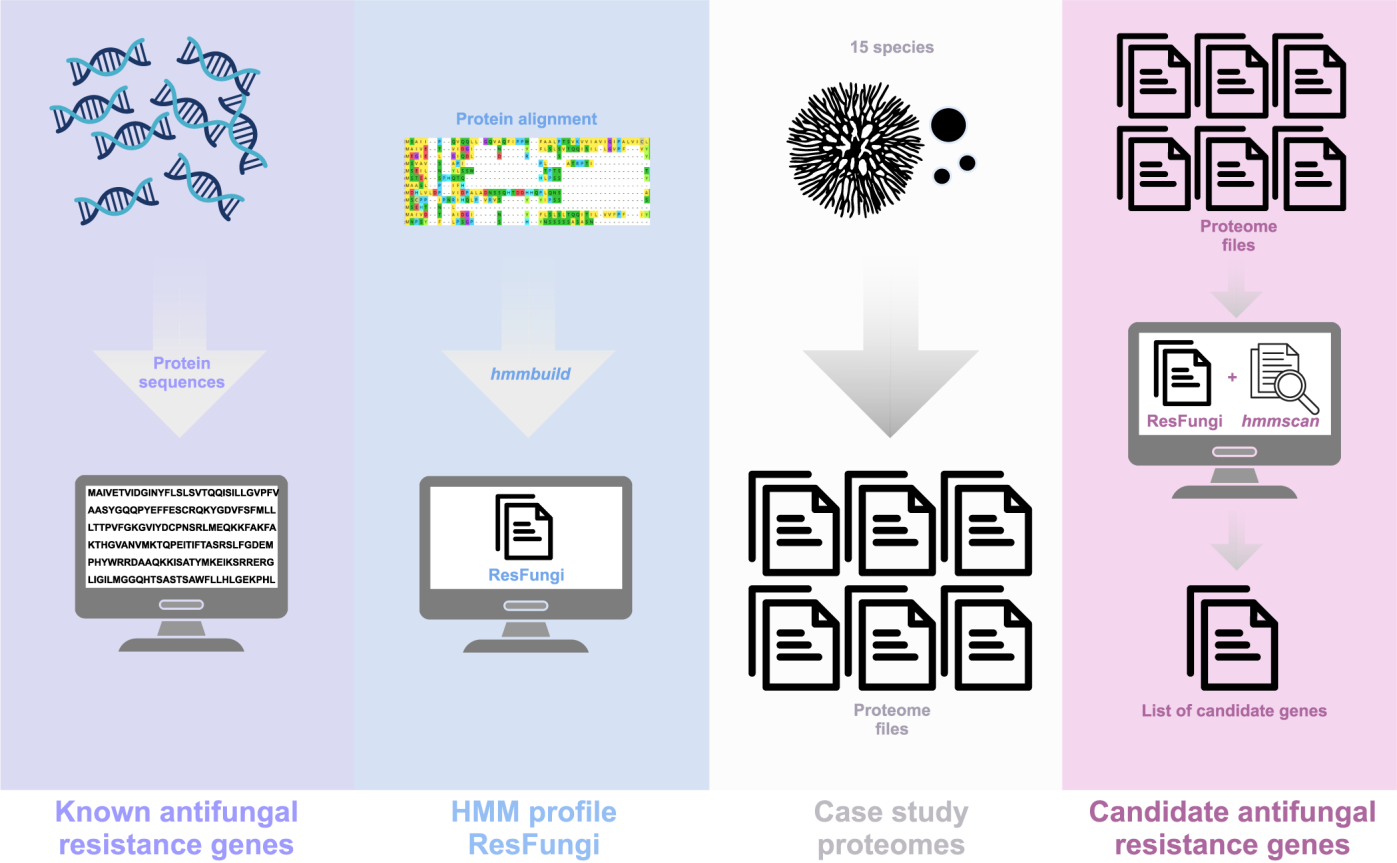
Step-by-step pipeline from the creation of the ResFungi HMM database to execute the analysis of the antifungal candidate genes.

### Searching Candidate Antifungal Resistance Genes

After the HMM protein database was generated, ResFungi was used to search for candidate antifungal resistance genes in the species aforementioned in a customized pipeline (Figure 1). HMMER3 was used to search for conserved protein domains and candidate antifungal resistance genes using the function *hmmscan*. Based on the gene hits found that were consistent with the mutation description in MARDy, a sequence score filter of 100 and a domain score filter of 50 were applied. Genes that had either one of these scores lower than the filters were discarded.

## Results

The MARDy database, accessed January 18th 2024, had 28 drugs with gene mutations described as conferring antifungal resistance, represented by 18 genes. Here, the drugs were only considered for analysis if at least three gene sequences with known resistance mutations were obtained. This step was performed to filter out drugs with unknown genes and/or very few sequences, avoiding unreliable results. After filtering those drugs with at least three sequences available for download, five drugs were added to ResFungi: fluconazole, itraconazole, voriconazole, micafungin, and caspofungin. A sixth category was created because some genes were classified as conferring resistance to azoles, without specifying which antifungal resistance from this class. For this reason, an HMM was created for each drug, with an additional HMM for azole-resistance genes. A seventh category was also added due to papers that have described genes responsible for flucytosine resistance.^26,27^ 18 genes found using MARDy, along with 38 genes described by dos Santos et al.,^29^ eight genes by Chang et al.,^27^ and eight genes by Rizzo et al.,^28^ compose ResFungi, which currently has 72 genes and seven HMMs.

**Figure 2:**
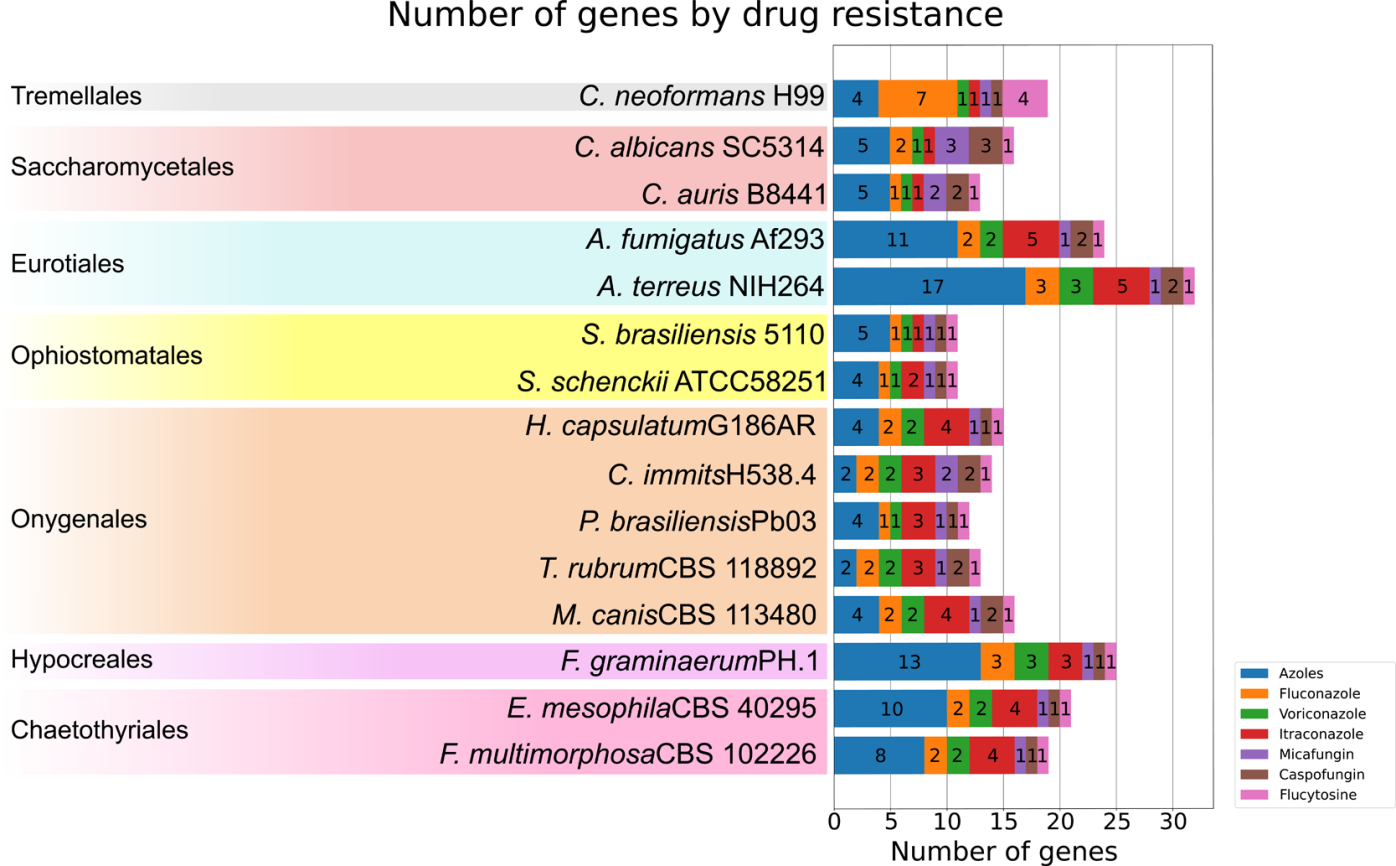
Summary of candidate drug resistance-related genes per drug. The species names belonging to the same fungal order are highlighted in the same color with the order name on the left side. The numbers inside the boxes with different colors represent the number of candidate genes found that could confer resistance to that drug.

The search for candidate genes related to drug resistance resulted in different numbers of genes for the 15 species used as case studies. The number of genes found varied for different drugs and species. No specific pattern was observed for species in the same genus or the same order (Figure 2). *Aspergillus terreus* was the species with the highest number of candidate genes found (32), while both species of *Sporothrix* had the lowest number of candidate genes (11). This result shows that the order Ophiostomatales had the least number of drug-resistant genes described so far. Despite that both *Sporothrix* species have the same number of candidate genes, *S. brasiliensis* had five azole-resistance genes and one for each of the other drugs, while *S. schenckii* had four azole-resistance genes, two caspofungin-resistance genes, and one for each of the other drugs. Although the number of candidate genes varies among species and orders, this result does not mean that other drug resistance-related genes are not present in these species, meaning that the list of candidate genes found was the ones ResFungi was able to retrieve at the moment (Table 1). Therefore, as other drug resistance genes become available in the literature, ResFungi will be updated.

**Table 1:** List of Candidate Antifungal Resistance Genes Found after HMM Analysis Using the ResFungi HMM Database^a^

strains tested	drug	gene ID	gene annotation
*Cryptococcus neoformans* H99	flucytosine	CNAG_00613	cytosine deaminase
		CNAG_02337	uracil phosphoribosyltransferase
		CNAG_03322	UDP-glucuronate decarboxylase
		CNAG_01681	cytosine permease
	azoles	CNAG_00730	ATP-binding cassette transporter
		CNAG_04098	ATP-binding cassette, subfamily G (WHITE), member 2, PDR
		CNAG_07799	ABC transporter
		CNAG_00869	ATP-binding cassette transporter
	caspofungin	CNAG_06508	1,3-beta-glucan synthase component FKS1
	fluconazole	CNAG_00040*	cytochrome P450, family 51 (sterol 14-demethylase)
		CNAG_05153	hypothetical protein
		CNAG_05222	transcriptional regulator Nrg1
		CNAG_01242	hypothetical protein
		CNAG_07680	HAP5
		CNAG_04263	hypothetical protein
		CNAG_06283	nucleolar protein
	itraconazole	CNAG_00040	cytochrome P450, family 51 (sterol 14-demethylase)
	micafungin	CNAG_06508	1,3-beta-glucan synthase component FKS1
	voriconazole	CNAG_00040	cytochrome P450, family 51 (sterol 14-demethylase)
*Candida albicans* SC5314	flucytosine	C5_03390C_A	uracil phosphoribosyltransferase
	azoles	C3_04070C_A	Cdr11p
		C3_04890W_A	multidrug resistance protein CDR2
		C1_08070W_A	Cdr4p
		C3_05220W_A	pleiotropic ABC efflux transporter of multiple drugs CDR1
		C6_03840C_A	ATP-binding cassette transporter
	caspofungin	C1_05600W_A*	1,3-beta-d-glucan-UDP glucosyltransferase
		CR_00850C_A	1,3-beta-d-glucan-UDP glucosyltransferase
		C1_02420C_A	1,3-beta-d-glucan-UDP glucosyltransferase
	fluconazole	C5_00660C_A *	lanosterol 14-alpha demethylase
		C2_06470W_A *	phospholipid-translocating ATPase
	itraconazole	C5_00660C_A *	lanosterol 14-alpha demethylase
	micafungin	C1_05600W_A	1,3-beta-d-glucan-UDP glucosyltransferase
		CR_00850C_A	1,3-beta-d-glucan-UDP glucosyltransferase
		C1_02420C_A	1,3-beta-d-glucan-UDP glucosyltransferase
	voriconazole	C5_00660C_A *	lanosterol 14-alpha demethylase
*Candida auris* B8441	flucytosine	B9J08_004076	uracil phosphoribosyltransferase activity
	azoles	B9J08_004452	hypothetical protein
		B9J08_000164	putative transmembrane xenobiotic transporter
		B9J08_002451	putative efflux transporter
		B9J08_000479	putative ABC transporter family transmembrane transporter
		B9J08_001125	hypothetical protein
	caspofungin	B9J08_001020	putative 1,3-beta-d-glucan synthase
		B9J08_000964*	putative 1,3-beta-d-glucan synthase
	fluconazole	B9J08_001448*	putative sterol 14-demethylase activity
	itraconazole	B9J08_001448	putative sterol 14-demethylase activity
	micafungin	B9J08_001020	putative 1,3-beta-d-glucan synthase
		B9J08_000964*	putative 1,3-beta-d-glucan synthase
	voriconazole	B9J08_001448	putative sterol 14-demethylase activity
*Aspergillus fumigatus* AF293	flucytosine	AFUA_5G05460	cytosine deaminase-uracil phosphoribosyltransferase fusion protein
	azoles	AFUA_4G01050	ABC multidrug transporter%2C putative
		AFUA_6G08020	ABC transporter%2C putative
		AFUA_1G14330	ABC transporter%2C putative
		AFUA_1G17440	ABC drug exporter AbcA
		AFUA_5G09460	ABC transporter%2C putative
		AFUA_3G07300	ABC multidrug transporter%2C putative
		AFUA_2G15130	ABC multidrug transporter%2C putative
		AFUA_6G04360	ABC drug exporter AtrF
		AFUA_5G02260	ABC multidrug transporter%2C putative
		AFUA_5G00790	ABC multidrug transporter%2C putative
		AFUA_3G01400	ABC multidrug transporter%2C putative
	caspofungin	AFUA_6G12400	1,3-beta-glucan synthase catalytic subunit FksP
		AFUA_4G08340	heme a biosynthesis protein%2C putative
	fluconazole	AFUA_7G03740	14-alpha sterol demethylase Cyp51B
		AFUA_4G06890	14-alpha sterol demethylase Cyp51A
	itraconazole	AFUA_6G05300	CCAAT-binding factor complex subunit HapE
		AFUA_7G03740	14-alpha sterol demethylase Cyp51B
		AFUA_2G01260	HLH transcription factor%2C putative
		AFUA_4G08340	heme a biosynthesis protein%2C putative
		AFUA_4G06890	14-alpha sterol demethylase Cyp51A
	micafungin	AFUA_6G12400	1,3-beta-glucan synthase catalytic subunit FksP
	voriconazole	AFUA_7G03740	14-alpha sterol demethylase Cyp51B
		AFUA_4G06890	14-alpha sterol demethylase Cyp51A
*Aspergillus terreus* NIH264	flucytosine	ATEG_09482	uracil phosphoribosyltransferase
	azoles	ATEG_10369	hypothetical protein
		ATEG_09933	hypothetical protein
		ATEG_09091	hypothetical protein
		ATEG_08878	ABC transporter CDR4
		ATEG_07864	ABC transporter CDR4
		ATEG_07017	hypothetical protein
		ATEG_07138	ABC transporter CDR4
		ATEG_07321	hypothetical protein
		ATEG_06319	hypothetical protein
		ATEG_06504	hypothetical protein
		ATEG_05640	ABC transporter CDR4
		ATEG_05854	hypothetical protein
		ATEG_03243	hypothetical protein
		ATEG_01482	hypothetical protein
		ATEG_00063	hypothetical protein
		ATEG_00304	hypothetical protein
		ATEG_00635	multidrug resistance protein CDR1
	caspofungin	ATEG_05362	conserved hypothetical protein
		ATEG_03278	1,3-beta-glucan synthase component GLS2
	fluconazole	ATEG_10302	cytochrome P450 51
		ATEG_05917	cytochrome P450 51
		ATEG_02850	cytochrome P450 51
	itraconazole	ATEG_10302	cytochrome P450 51
		ATEG_08156	conserved hypothetical protein
		ATEG_05632	nuclear transcription factor Y subunit C-2
		ATEG_05917	cytochrome P450 51
		ATEG_02850	cytochrome P450 51
	micafungin	ATEG_03278	1,3-beta-glucan synthase component GLS2
	voriconazole	ATEG_10302	cytochrome P450 51
		ATEG_05917	cytochrome P450 51
		ATEG_02850	cytochrome P450 51
*Sporothrix brasiliensis* 5110	flucytosine	SPBR_07698	uracil phosphoribosyltransferase
	azoles	SPBR_06357	ABC drug exporter AtrF
		SPBR_02187	ABC transporter CDR4
		SPBR_04049	ATP-binding protein cassette transporter
		SPBR_05490	ABC multidrug transporter
		SPBR_05469	hypothetical protein
	caspofungin	SPBR_04029	1%2C3-beta-glucan synthase
	fluconazole	SPBR_08369	cytochrome P450%2C family 51
	itraconazole	SPBR_08369	cytochrome P450%2C family 51
	micafungin	SPBR_04029	1%2C3-beta-glucan synthase
	voriconazole	SPBR_08369	cytochrome P450%2C family 51
*Sporothrix schenckii* ATCC58251	flucytosine	HMPREF1624_00629	uracil phosphoribosyltransferase
	azoles	HMPREF1624_07625	hypothetical protein
		HMPREF1624_04293	hypothetical protein
		HMPREF1624_04362	hypothetical protein
		HMPREF1624_02339	hypothetical protein
	caspofungin	HMPREF1624_08332	1,3-beta-glucan synthase component FKS1
	fluconazole	HMPREF1624_01477	hypothetical protein
	itraconazole	HMPREF1624_02728	transcriptional activator HAP5
		HMPREF1624_01477	hypothetical protein
	micafungin	HMPREF1624_08332	1,3-beta-glucan synthase component FKS1
	voriconazole	HMPREF1624_01477	hypothetical protein
*Histoplasma capsulatum* G186AR	flucytosine	HCBG_02188	uracil phosphoribosyltransferase
	azoles	HCBG_08663	ABC drug exporter AtrF
		HCBG_07766	ABC transporter
		HCBG_04961	ABC transporter
		HCBG_00215	ABC transporter CDR4
	caspofungin	HCBG_02657	glucan synthase
	fluconazole	HCBG_09081	lanosterol 14-alpha-demethylase
		HCBG_00405	cytochrome P450 sterol 14 alpha-demethylase
	itraconazole	HCBG_09081	lanosterol 14-alpha-demethylase
		HCBG_08879	CCAAT-binding factor complex subunit HapE
		HCBG_03969	protoheme IX farnesyltransferase
		HCBG_00405	cytochrome P450 sterol 14 alpha-demethylase
	micafungin	HCBG_02657	glucan synthase
	voriconazole	HCBG_09081	lanosterol 14-alpha-demethylase
		HCBG_00405	cytochrome P450 sterol 14 alpha-demethylase
*Coccidioides immitis* H538.4	flucytosine	CIHG_00729	uracil phosphoribosyltransferase
	azoles	CIHG_02380	pleiotropic ABC efflux transporter of multiple drugs
		CIHG_09915	ABC transporter
	caspofungin	CIHG_05825	1,3-beta-glucan synthase component GLS2
		CIHG_05826	1,3-beta-glucan synthase component bgs2
	fluconazole	CIHG_02429	cytochrome P450 51
		CIHG_09980	cytochrome P450 51
	itraconazole	CIHG_02429	cytochrome P450 51
		CIHG_04463	nuclear transcription factor Y subunit C-4
		CIHG_09980	cytochrome P450 51
	micafungin	CIHG_05825	1,3-beta-glucan synthase component GLS2
		CIHG_05826	1,3-beta-glucan synthase component bgs2
	voriconazole	CIHG_02429	cytochrome P450 51
	voriconazole	CIHG_09980	cytochrome P450 51
*Paracoccidioides brasiliensis* Pb03	flucytosine	PABG_05584	uracil phosphoribosyltransferase
	azoles	PABG_01584	hypothetical protein
		PABG_03691	hypothetical protein
		PABG_05655	hypothetical protein
		PABG_12450	hypothetical protein
	caspofungin	PABG_04524	1,3-beta-glucan synthase component FKS1
	fluconazole	PABG_01406	hypothetical protein
	itraconazole	PABG_00651	protoheme IX farnesyltransferase
		PABG_01406	hypothetical protein
		PABG_12156	transcriptional activator HAP5
	micafungin	PABG_04524	1,3-beta-glucan synthase component FKS1
	voriconazole	PABG_01406	hypothetical protein
*Trichophyton rubrum* CBS 118892	flucytosine	TERG_04475	hypothetical protein
	azoles	TERG_02508	hypothetical protein
		TERG_04227	hypothetical protein
	caspofungin	TERG_01127	1,3-beta-glucan synthase component FKS1
		TERG_05496	protoheme IX farnesyltransferase
	fluconazole	TERG_01703	hypothetical protein
		TERG_02984	hypothetical protein
	itraconazole	TERG_01703	hypothetical protein
		TERG_02984	hypothetical protein
		TERG_05496	protoheme IX farnesyltransferase
	micafungin	TERG_01127	1,3-beta-glucan synthase component FKS1
	voriconazole	TERG_01703	hypothetical protein
		TERG_02984	hypothetical protein
*Microsporum canis* CBS 113480	flucytosine	MCYG_01927	uracil phosphoribosyltransferase
	azoles	MCYG_01980	ATP-binding cassette transporter
		MCYG_06743	brefeldin A resistance protein
		MCYG_07449	ABC transporter
		MCYG_08227	ABC transporter
	caspofungin	MCYG_02678	protoheme IX farnesyltransferase
		MCYG_03244	1,3-beta-glucan synthase component GLS2
	fluconazole	MCYG_07010	cytochrome P450 51
		MCYG_07307	cytochrome P450 51
	itraconazole	MCYG_02678	protoheme IX farnesyltransferase
		MCYG_05272	CCAAT-binding factor complex subunit HapE
		MCYG_07010	cytochrome P450 51
		MCYG_07307	cytochrome P450 51
	micafungin	MCYG_03244	1,3-beta-glucan synthase component GLS2
	voriconazole	MCYG_07010	cytochrome P450 51
		MCYG_07307	cytochrome P450 51
*Fusarium graminearum* PH.1	flucytosine	FGSG_07475	uracil phosphoribosyltransferase
	azoles	FGSG_04580	hypothetical protein
		FGSG_03882	hypothetical protein
		FGSG_03735	hypothetical protein
		FGSG_02870	hypothetical protein
		FGSG_02847	hypothetical protein
		FGSG_07383	hypothetical protein
		FGSG_08830	hypothetical protein
		FGSG_08312	hypothetical protein
		FGSG_08309	hypothetical protein
		FGSG_09329	hypothetical protein
		FGSG_10577	hypothetical protein
		FGSG_11272	hypothetical protein
		FGSG_11240	hypothetical protein
	caspofungin	FGSG_07946	1,3-beta-glucan synthase component GLS2
	fluconazole	FGSG_01000	cytochrome P450 51
		FGSG_04092	cytochrome P450 51
		FGSG_11024	cytochrome P450 51
	itraconazole	FGSG_01000	cytochrome P450 51
		FGSG_04092	cytochrome P450 51
		FGSG_11024	cytochrome P450 51
	micafungin	FGSG_07946	1,3-beta-glucan synthase component GLS2
	voriconazole	FGSG_01000	cytochrome P450 51
		FGSG_04092	cytochrome P450 51
		FGSG_11024	cytochrome P450 51
*Exophiala mesophila* CBS 40295	flucytosine	PV10_05542	uracil phosphoribosyltransferase
	azoles	PV10_09203	hypothetical protein
		PV10_08426	hypothetical protein
		PV10_06197	hypothetical protein
		PV10_04312	hypothetical protein
		PV10_02380	hypothetical protein
		PV10_02898	hypothetical protein%2C variant
		PV10_03185	hypothetical protein
		PV10_00779	hypothetical protein
		PV10_01361	hypothetical protein%2C variant
		PV10_01362	hypothetical protein
	caspofungin	PV10_00705	1,3-beta-glucan synthase component FKS1
	fluconazole	PV10_06729	hypothetical protein
		PV10_02323	hypothetical protein
	itraconazole	PV10_05519	hypothetical protein
		PV10_06729	hypothetical protein
		PV10_01998	protoheme IX farnesyltransferase
		PV10_02323	hypothetical protein
	micafungin	PV10_00705	1,3-beta-glucan synthase component FKS1
	voriconazole	PV10_06729	hypothetical protein
		PV10_02323	hypothetical protein
*Fonsecaea multimorphosa* CBS 102226	flucytosine	Z520_12305	hypothetical protein
	azoles	Z520_11453	hypothetical protein
		Z520_11424	hypothetical protein
		Z520_05127	hypothetical protein
		Z520_04261	hypothetical protein
		Z520_04553	hypothetical protein
		Z520_01984	hypothetical protein
		Z520_01538	hypothetical protein
		Z520_00463	hypothetical protein
	caspofungin	Z520_01031	1,3-beta-glucan synthase component FKS1
	fluconazole	Z520_06611	hypothetical protein
		Z520_01512	hypothetical protein
	itraconazole	Z520_08373	hypothetical protein
		Z520_06611	hypothetical protein
		Z520_06203	hypothetical protein
		Z520_01512	hypothetical protein
	micafungin	Z520_01031	1,3-beta-glucan synthase component FKS1
	voriconazole	Z520_06611	hypothetical protein
		Z520_01512	hypothetical protein

a Genes marked with ‘*’ were already described in MARDy as conferring antifungal resistance to the drug found in this analysis

The list of candidate genes comprises 261 genes, with 90 (34.48%) of them annotated as hypothetical proteins. This means that over 65% of the candidate genes have functional annotations. Such a result allowed the investigation and validation of these genes as possibly being involved in antifungal resistance to different drugs. For instance, 10 of the 15 species, either the ERG11 or the CYP51 genes, encoding proteins of the ergosterol biosynthesis pathway, were retrieved as conferring resistance to at least one of the azoles (fluconazole, itraconazole, and voriconazole) tested. This finding is consistent with previous studies.^3,4,32^ Interestingly, ResFungi returned several transporter coding genes as candidates for azole resistance (Table 1). This mechanism has been described in several fungi, such as *Candida* spp., *Cryptococcus* spp., *Aspergillus* spp.,^33^ and *Fusarium* spp.^34^ Besides the azole resistance genes, FKS genes that encode glucan synthase were retrieved as candidates for micafungin and caspofungin resistance. These findings are also consistent with the literature.^18,35 −37^

The genes with known antifungal resistance related to flucytosine were obtained from papers that studied resistance to this drug in cryptococcii, as other genes from other fungal species were not available at the time this database was created. Despite this limitation, candidate genes were found in the other species tested here. As expected, all four genes related to flucytosine resistance in *Cryptococcus* were retrieved in this analysis: cytosine deaminase (Fcy1), uracil phosphoribosyltransferase (Fur1), UDP-glucuronate decarboxylase (Uxs1), and cytosine permease (Fcy2). Surprisingly, all of the other species showed the Fur1 gene as a candidate for flucytosine resistance. This result showed that even though no gene belonging to a representative of the other species was sampled as being related to flucytosine resistance, the ResFungi analysis was still able to capture a candidate gene for such drug resistance. Furthermore, the genes retrieved in the noncryptococcii species have the same annotation as Fur1 in *C. neoformans*, which provides more confidence for the results found. Interestingly, the candidate gene found in *A. fumigatus* is annotated as a protein fusion between cytosine deaminase and uracil phosphoribosyltransferase (Fcy1 and Fur1, respectively). Such finding helps in formulating new hypotheses to be tested in terms of the origin of the flucytosine resistance mechanism in *A. fumigatus* or if such gene indeed confers flucytosine resistance to it.

In addition to retrieving known genes related to drug resistance, ResFungi also showed genes that have not yet been described as being related to antifungal resistance in different fungal species. The first example is the list of candidate genes found for *C. neoformans*. The only gene with a known role in antifungal resistance found in MARDy is ERG11 (Table 1). As of the time of this study, no genes had been described in *Cryptococcus* spp. that could be related to micafungin/caspofungin resistance; however ResFungi results were able to retrieve a candidate gene in *C. neoformans*. The candidate gene is annotated as FKS1, which has been described as being involved in micafungin/caspofungin resistance in other fungal species.^29,32^

Another example of candidate genes without a known antifungal resistance role in a species is the list for *Candida* species. In *C. albicans*, two different genes were found for caspofungin resistance, with one of them already known, but the other was a novel prediction finding (Table 1). The functional annotation of both genes is the same, which makes a stronger case for the candidate gene being, in fact, related to caspofungin resistance as well. A similar scenario was observed when analyzing *C. auris* genes. A fluconazole resistance gene was also a hit for voriconazole and itraconazole resistance (Table 1). This gene is annotated as ERG11, which is described in the MARDy database^18^ as conferring resistance to fluconazole, voriconazole, and itraconazole in *C. albicans*. The same scenario was found for micafungin and caspofungin resistance genes (Table 1). The previously described gene related to resistance to caspofungin and micafungin is annotated as FKS1. The candidate gene found for the same drugs is annotated as FKS2, which has been described as related to micafungin resistance in *Candida glabrata*.^32^

The number of candidate genes found for each of the drugs analyzed highlights the differences among the groups of drugs. The drug category with the highest number of candidate genes found is “azoles” (genes with azole resistance described without specifying the type of azole). This number is even higher when adding the candidate genes conferring resistance to fluconazole, itraconazole, and voriconazole, representing 77% of the entire gene list. The remaining 23% represent candidate genes that confer resistance to micafungin, caspofungin, and flucytosine, where the latter has the lowest number of candidate genes found. The discrepancy in the number of candidate genes found for azole resistance and the other drugs highlights the importance of conducting additional studies to identify candidate genes associated with resistance to micafungin, caspofungin, and flucytosine.

## Discussion

Antifungal resistance profile HMMs are still unavailable, which partially explains the difficulty in adding more drugs to ResFungi. Another challenge we faced during the database construction was that some sequences were not available at NCBI anymore, or fewer than three sequences were retrieved for a certain drug. As more genes are described as conferring antifungal resistance, these data will be added to ResFungi. Moreover, genome-based predictors for antifungal resistance are still unavailable,^8^ while such predictors are widely used for microbial resistance.^24,25^ Therefore, ResFungi is not only a pioneer database for future studies and databases but allows the description of candidate antifungal resistance genes in species without prior resistance studies.

The well-known HMM profiles can be found for antibacterial resistance genes, such as Resfams^24^ and AMRFinderPlus,^25^ but such profiles are unavailable for antifungal resistance genes. Although these databases can be helpful to understand the mechanisms behind drug resistance, such databases do not allow specific insights into antifungal drugs or genes related to resistance to these specific drugs. Therefore, the construction of an HMM profile, such as ResFungi, is necessary for rational studies on antifungal resistance genes, since in silico approaches can reduce the time to find gene candidates, narrow down the number of candidate genes, and thus, lower costs for wet lab experiments.

Although ResFungi represents an important step toward learning more about antifungal resistance, the scarce number of gene mutations leading to antifungal resistance is still a limitation to the number of drugs present in ResFungi. Mutations conferring antifungal resistance are often described in drug targets, which is also a limitation for the creation of databases. Despite the limitations mentioned, the use of ResFungi helps unravel resistance mechanisms present in fungal species. For instance, the enzyme “protoheme IX farnesyltransferase” was retrieved in both *T. rubrum* and *E. mesophila*. A putative farnesyltransferase has been described as conferring resistance to itraconazole and caspofungin^29^ in *A. fumigatus*. The same enzyme seems to confer resistance to both drugs in *T. rubrum*, while the enzyme appears to only confer resistance to itraconazole in *E. mesophila*. The identification of this mechanism and the type of resistance it might be involved in allow future research on drugs targeting this specific mechanism to stop the resistance to itraconazole and caspofungin in those species.

The genetic mechanism of the flucytosine (FC) resistance pathway involves three main enzymes described in the literature so far: transport of FC into the cell through cytosine permease, deamination of FC to 5-fluorouracil (5-FU) by cytosine deaminase, and conversion of 5-FU to 5-fluorouridine monophosphate. The subsequent steps of the mechanism lead to DNA and protein synthesis disruption.^38^ In our results, at least one of these enzymes were retrieved as being involved in FC resistance in the species studied. In *A. fumigatus*, the enzyme found is annotated as the “cytosine deaminase-uracil phosphoribosyltransferase fusion protein”.

Azole resistance mechanisms (including fluconazole, voriconazole, and itraconazole) described so far involve mutations in the lanosterol 14-α-demethylase (ERG11) gene. ERG11 mutations lead to ergosterol biosynthesis blockage and accumulation of 14-α-methyl-3,6-diol, a toxic intermediate product of the ergosterol pathway.^33^ Among the results for candidate genes related to azole resistance, at least one copy of lanosterol 14-α-demethylase was found. Besides the presence of lanosterol 14-α-demethylase genes, genes related to the ABC-type transporter activity are also involved in a mechanism of azole resistance that upregulates the efflux pump.^33^ In *C. neoformans*, another resistance mechanism has been described involving extracellular vesicle production and cellular lipid homeostasis, where the transcription factor HAP5 has been described as having an important role in the mechanism.^28^ In our results, other subunits of the HAP transcription factor have been found in the following species: *A. fumigatus*, *S. schenckii*, *H. capsulatum*, *P. brasiliensis*, and *M. canis*. The presence of this transcription factor suggests that a similar mechanism is present in the aforementioned species.

The main mechanism of echinocandin (caspofungin and micafungin) resistance inhibits the (1,3)-β-d-glucan synthase, causing cell wall disruption and severe stress. The glucan synthase is encoded by the Fks1 and Fks2 genes, where mutations in these genes might lead to micafungin/caspofungin resistance.^33^ Genes enconding the (1,3)-β-d-glucan synthase were found in all species studied here, although a difference in the copy number has been identified. The following species had more than one copy retrieved: *Candida albicans* with three copies; *C. auris*, *A. fumigatus*, *A. terreus,* and *C. immitis* with two copies each. In *A. fumigatus*, heme A biosynthesis proteins have been described to have an important role in multidrug resistance.^29^ These proteins provide stability and folding of the Cox1 subunit, where deficiencies in this complex lead to mitochondrial dysfunction. Heme A proteins have also been retrieved in *A. fumigatus*, *H. capsulatum*, *M. canis*, *T. rubrum,* and *P. brasiliensis* as candidate genes conferring resistance to both micafungin and itraconazole.

Drug efflux is a mechanism widely studied and documented as an antimicrobial resistance mechanism. In fungi, the major facilitator superfamily (MFS), ATP-binding cassette (ABC) transporters, *Candida* drug resistance 1 and 2 (Cdr1 and Cdr2), and multidrug resistance 1 (Mdr1) have been associated with fluconazole resistance.^2,33,39^ However, the relevance of transporters in azole resistance has not yet been described in *Sporothrix* species. These findings allow for further research about the impact of transporters in antifungal resistance, especially in species where such mechanism has not been yet documented.

Flucytosine resistance and its mechanism of action are mainly studied in *Candida*, *Cryptococcus,* and *Aspergillus* species. Among the candidate genes found by ResFungi analysis, the FUR1 gene, described as flucytosine-related resistance in *Candida*,^40,41^ has been retrieved in this study. In *Aspergillus*, resistance to flucytosine was linked to mutants of the FCYB gene depending on the pH of the growth media.^42^ On the other hand, ResFungi analysis pointed to FUR1 as a candidate gene possibly related to flucytosine resistance, which can be tested in future studies.

As of the moment, hypothetical proteins were retrieved as related to candidate genes that could confer resistance to the drugs tested here. Although these proteins were not assigned a functional role in these species, the results obtained here help narrow the possible functions of these genes in wet lab experiments. At the moment, the limited number of antifungal resistance genes available represents a challenge to measure and assess the number of false positives; we hope ResFungi helps increase the amount of studies about antifungal resistance genes. Also, the results found indicate that the use of ResFungi in future research focusing on different fungal species can help elucidate antifungal drug resistance mechanisms in species with little to no prior knowledge of such mechanisms. In addition to finding antifungal resistance candidate genes, ResFungi allows describing probable molecular mechanisms involved in different drugs and species. Azole resistance can be related to several mechanisms,^2^ some of which is shown by the candidate genes found after ResFungi analysis (Table 1). Many molecular mechanisms involved in different drug resistance in *Sporothrix* are still poorly known.^43^ Also, *Sporothrix globosa* was described as more sensitive to both micafungin and caspofungin during the yeast phase.^44^ Our results show the beta-glucan synthesis pathway as a candidate mechanism involved in such susceptibility in other *Sporothrix* species (Table 1). Furthermore, up until now, there have been no clinical breakpoints or epidemiological cutoff values to establish resistance or susceptibility to antifungals for several species. Thus, as more studies address these issues, the need for a resistance database has become more prominent. Despite protein profile HMMs being widely used for finding antibacterial resistance genes, there is a lack of such profiles for antifungal genes. Antibacterial HMM databases are well established and composed of over 600 genes related to different classes of antimicrobial resistance, which enables the description of bacterial resistomes.^24,25,45^

## Conclusions

The database presented in this work allows the construction of protein profile HMMs specific to antifungal resistance genes and the possibility of helping to describe the antifungal resistome of fungal species. To summarize, ResFungi showed promising results in finding known genes related to antifungal drug resistance and identifying candidate genes for resistance to other drugs. Therefore, ResFungi is a powerful tool to help narrow down candidate genes related to fungal drug resistance and may also be useful for the rational design of experiments aiming to address the genetic basis of antifungal resistance.

## Data Availability

ResFungi is available at https://github.com/deCarvalho90/ResFungi.
